# Optimization of workflow and screening panels for the detection of malignant monoclonal gammopathies

**DOI:** 10.1515/almed-2020-0042

**Published:** 2020-07-27

**Authors:** Raquel Oliveros Conejero, Pilar Pascual Usandizaga, Adolfo Garrido Chércoles

**Affiliations:** Department of Clinical Biochemistry, Hospital Universitario Donostia, San Sebastián, Guipúzcoa, Spain

**Keywords:** multiple myeloma, screening algorithm, workload

## Abstract

**Objectives:**

Multiple myeloma (MM) is one of the hematologic malignancies with a greater delay in diagnosis. Laboratories receive numerous MM screening test requests without a specific suspicion, which entails a substantial workload and reduces the efficiency of laboratories. Objective: to increase the efficacy of MM screening protocols.

**Methods:**

The results of serum protein electrophoresis (SPEP), serum protein immunofixation electrophoresis (SIFE), urine protein immunofixation electrophoresis, and serum free light chain assays of 75 patients with MM, three with amyloidosis, and a patient with solitary plasmocytoma were collected. The frequency of a set of biochemical alterations in these patients was compared with that in controls (n=120). A validation of the screening algorithm was carried out in 261 consecutive patients with a clinical or analytical suspicion of MM.

**Results:**

SPEP+SFLC or SIFE+SFLC (98% sensitivity) were the screening algorithms with the highest sensitivity. Prospectively, the SPEP + SFLC detected 27 of the 28 confirmed cases of MG and saved 15 h of work. Alterations in five of the six parameters studied were more frequent in the study group, with a cumulative value of ≥3 parameters altered (61.1 vs. 1.7%) (positive predictive value: 85%; negative predictive value: 94%).

**Conclusions:**

The SPEP+SFLC screening protocol demonstrated the highest sensitivity and was the least time-consuming protocol. In addition, this protocol improves diagnostic sensitivity and laboratory performance.

## Introduction

Multiple myeloma (MM) is a clonal disorder of plasma cells characterized by bone marrow plasmacytosis and, in most cases, the production of an abnormal monoclonal immunoglobulin that is detectable by protein electrophoresis in serum and/or urine. The release of neoplastic plasma cells and monoclonal immunoglobulin induces organ and tissue damage resulting in the development of the symptoms typically associated with MM, including bone lesions (which manifest in the form of bone pain, pathological fractures, and osteolysis, to name a few); anemia, hypercalcemia, and kidney damage. National and international guidelines have been published for the diagnosis and monitoring of patients with multiple myeloma [1], [2], [3].

Serum protein electrophoresis (SPE) is the most widely used technique for the detection of monoclonal gammopathies. However, the sensitivity of this test is limited, especially if the monoclonal component is small or only contains light chains [4]. For this reason, on suspicion of a monoclonal gammopathy (MG), the International Myeloma Working Group (IMWG) recommends the use of a battery of serum screening tests for the detection of monoclonal proteins (MP), which includes SPE, serum immunoglobulin-free light chain (FLC) testing, and serum immunofixation electrophoresis (IFE) [1]. This screening algorithm has a high diagnostic sensitivity and specificity, and only requires a sample of serum, which spares the patient and the laboratory the inconveniences of collecting and processing a 24-h urine sample. Notwithstanding the simplicity of the recommended algorithm, a high volume of requests for serum and urine proteinograms are received in our center without an indication of suspected monoclonal gammopathy. This situation makes it difficult to decide whether the screening test recommended by the IMWG should be applied. However, the use of a single screening test can yield false negative results and delay diagnosis, with an impact on survival time and/or the development of severe comorbidities [5].

In this scenario, this study has a two-fold objective: to define a profile for suspicion of MG based on (i) laboratory test results (anemia, hypercalcemia, hyperproteinemia, increased creatinine, immunoparesis); (ii) bone pain; and (iii) an abnormal serum protein electrophoresis. Another objective is to identify the most cost-effective screening techniques to avoid the unnecessary use of a large and complex panel that extends the turnaround time.

## Materials and methods

### A retrospective study

A retrospective study was conducted of data of 79 patients, of whom 75 had a diagnosis of multiple myeloma (MM), 3 had amyloidosis (AL) and 1 had solitary plasmacytoma (SP). Diagnoses had been established between 2011 and 2014. Data were retrieved from the laboratory database (OMEGA 3000) and electronic medical records (Clinic). Data of 120 control patients without monoclonal gammopathy were also reviewed. The following data obtained within 30 days of diagnosis of MM/AL/SP prior to initiation of treatment were collected: results of serum immunofixation (SIFE), urine electrophoresis (UIFE), and serum free light chain determination (SFLCA); and signs suggestive of myeloma, namely: serum calcium (Ca)>10.2 mg/dL (reference rage [RR]: 8.8–10.2); creatinine (Cr)>1.8 mg/dL (RR: 0.4–1) or MDRD-4<60; Anemia (Hb<12 g/dL in women and Hb<13 g/dL in men); immunoparesis (IP) (IgG<7 g/L, RR: 7–16; IgA<0.7 g/L, RR: 0.7–4; IgM<0.4 g/L, RR: 0.4–2.3); hyperproteinemia (PT)>8.7 g/dL (RR: 6.6–8.7); and bone involvement. The period of 30 days was set to ensure that the results of the screening tests performed for differential diagnosis were available.

The reference ranges provided by the manufacturers were adopted for the purposes of the study.

### Prospective study

For the prospective study, the same data as those reviewed in the retrospective study (Ca, Hb, PT, Cr, MDRD-4, inmunoparesis and bone pain) were collected of all patients suspected to have a monoclonal gammopathy or whose physician had ordered a SPE, SIFE or UIFE test over a one-month period (October 2016).

### Methods

SPE was performed on a Capillarys 2 system, whereas SIFE and UIFE were undertaken on Hydrasys (Sebia) with the antisera provided by the manufacturer. Determination of serum immunoglobulin-free light chains was carried out using the SPAplus system and the Freelite reagent (The Binding Site). The normal range for serum free light chain *κ/λ* ratio was set at 0.26–1.65 [6]. In patients with a decreased glomerular filtration rate (estimated using the Modification of Diet in Renal Disease formula) identified by a MDRD <40, the normal range for the light chain *κ/λ* ratio was set at 0.37–3.1, as this improved the specificity of the assay [7].

General biochemistry determinations (calcium, total proteins, creatinine, and immunoglobulins) were performed on a COBAS c702 system (ROCHE), whereas hemoglobin was measured on a SYSMEX XN system (ROCHE).

### Calculation of turnaround time by technique

The following data were collected in relation to the technique used for suspicion of monoclonal gammopathy;

1. Turnaround time.
2. Time devoted exclusively to each technique.
3. Time devoted to validating test results.

### Statistical analysis

A study was performed to determine the sensitivity of each test used alone and in combination with another test for the detection of malignant MG (SPE, SIFE, UIFE, SFLCA) and identify the technique with the highest diagnostic sensitivity.

The positive predictive values (PPV) of each individual biochemical alteration and the number of alterations found to predict significant differences with respect to the control group was calculated.

Associations between categorical variables were assessed by Fisher’s exact test, and PPV and negative predictive values (NPV) were estimated with their 95% confidence intervals (CI). Data analysis was performed using MedCalc 10 and Excel.

## Results

### Retrospective analysis of the MP study: diagnostic sensitivity

The clinical and laboratory data of 79 patients with a confirmed diagnosis of multiple myeloma (MM), amyloidosis (AL), or solitary plasmacytoma (SP) and of 120 subjects without a MG diagnosis were reviewed to draw a profile of patients with suspicion of MG. The results of the four MP detection tests (SPE, SIFE, SFLCA, UIFE) and laboratory values suggestive of the disease (listed in the Materials and Methods section) were available at diagnosis in 54 of the 79 patients in the study group. These values were used to assess the sensitivity of each assay for the detection of MP when used alone and in combination with other tests (Table 1). The remaining 25 patients were excluded from analysis as their data were not valid for comparative analysis of diagnostic sensitivity.

**Table 1:** Sensitivity of the different test combinations for the identification of the monoclonal protein in patients diagnosed with MM (52), AL (1), or plasmacytoma (1).

Profile	Sensitivity, %			
	Only	SPEP	SIFE	UIFE
SFLC+	91	98	98	91
SPEP+	80		93	94
SIFE+	93			94
UIFE+	78			
SPEP + SFLC+			98	98
SPEP + SIFE+				94

SFLC, serum free light chain; SPEP, serum protein electrophoresis; SIFE, serum protein immunofixation electrophoresis; UIFE, urine protein immunofixation electrophoresis.

In this study group, the assays with the highest sensitivity were SIFE and SFLCA, with SPE or Bence Jones protein by UIFE having the lowest sensitivity. Sensitivity for the detection of MP reached 98% with the combination of several assays. This sensitivity was only achieved when SFLCA results were combined with those of another serum test. If SFLCA result was missing, maximum sensitivity did not exceed 94% even though UIFE results were included. The only case that was not detected by the serum-test-based protocol was a patient with non-secreting plasmacytoma, which was not detected by any of the four tests.

### Retrospective analysis of the MP study: profiles with reported suspicion of malignant MG

A retrospective analysis of all confirmed cases of MG and controls (patients without evidence of MG) was performed to assess the incidence of biochemical alterations in each group and identify potential biomarkers of MG. The identification of MG-specific biomarkers would be useful to raise suspicion and support the indication of screening for MP. Apart from levels of calcium, serum creatinine, total immunoglobulins (IgG, IgA, or IgM), hemoglobin, and total serum proteins, bone lesions at presentation were also included, as they has been described as the most common CRAB symptom (hypercalcemia, renal insufficiency, anemia, and bone lesions) in MM [8].

Evidence has been published that, except for hypercalcemia and renal insufficiency, patients with a diagnosis of malignant MG exhibit alterations in all the parameters studied (described in the Materials and Methods section), as compared to controls (Table 2). However, only TP values >8.7 g/dL and bone injury yielded a predictive positive value >80%: 100% (95% CI=82–100%) and 85% (95% CI=66–96%), respectively. As many as 43.3% of controls exhibited at least one of the signals or symptoms studied, although only 1.7% had three or more parameters altered. In contrast, the percentage of subjects in the MM group with alterations in three or more variables reached 61%.

**Table 2:** Frequency of alterations in the variables studied in the control and diseased group.

	Control 120,	MM/AL 54,	PPV%	NPV%	SEN%	SPE%
	n (%)	n (%)	(IC 95%)			
(A) Altered variable						
Hyperproteinemia	0 (0)	19 (35)	100	77	35	100
			(82–100)	(70–84)	(23–49)	(97–100)
Hypercalcemia	9 (7)	9 (16)	50	71	17	92
			(26–74)	(63–78)	(8–29)	(86–96)
Anemia	27 (22)	41 (76)	60	88	76	77
			(48–72)	(80–93)	(62–86)	(69–85)
Renal function deterioration	11 (9)	12 (22)	52	72	22	91
			(31–73)	(64–79)	(12–36)	(84–95)
Immunoparesia	18 (15)	43 (79)	70	90	79	85
			(57–81)	(83–95)	(66–89)	(77–91)
Bone involvement	4 (3)	23 (43)	85	78	43	97
			(66–96)	(71–85)	(29–57)	(92–99)
(B) Altered variables per patient						
0	68 (57)	0 (0)				
≥1	52 (43)	54 (100)	49	100		
			(41–61)	(95–100)		
≥2	15 (12)	43 (80)	74	90	80	87
			(61–85)	(84–95)	(66–89)	(80–93)
≥3	2 (2)	33 (61)	94	85	61	98
			(81–99)	(78–90)	(47–74)	(94–100)

n, number of patients; PPV, positive predictive value; NPV=negative predictive value; SEN=sensitivity; SPE=specificity.

The Table shows the percentage of patients without alterations in any variable and with concurrent alterations in 1, 2, 3 or more variables. PPV and NPV were calculated for each subgroup with respect to the total number of patients.

### Calculation of the time devoted to each MP screening test

The historical data of our laboratory reveal that the mean number of requests for SPE is 3180, 343 for SIFE, and 442 for UIFE per month. Our laboratory is fitted with four machines to perform these assays. An estimation was performed of the approximate time it takes to perform and validate each test:

1. SIFE=75 min for a plate of four samples (including dilution, application, migration, and staining).
2. UIFE=75 min for a plate of nine samples (including dilution, application, migration, and staining).
3. Electrophoresis=8 samples/hour (h).
4. Test validation: 45/hour.

Considering that there are 22 working days in a month, the mean time devoted daily to each assay is:

1. SIFE=4.9 h/day (343 requests/4 samples per plate × 75 min per plate/22 days).
2. UIFE=2.79 h/day (442 requests/9 samples per plate × 75 min per plate/22 days).
3. SPE=1.8 h/day (3180/80 samples per hour/22 days).
4. Test validation=4 h/day ( 3965 tests/45 tests per hour/22 days).

These tests take a total of 13.49 h/day (resulting from the addition of points 1–4). Up to 20, 50, and 25% of the requests for SPE, SIFE, and UIFE, respectively, corresponded to new patients (i.e., patients without a previous diagnosis of MG). Therefore, 31.5% of the time devoted to these assays corresponds to studies in new patients. In these patients, negative results accounted for 88% of SPE results, 73% of SIFE results, and 93% of UIFE results.

### Prospective performance study of the SPE + SFLCA algorithm: sensitivity and duration

**Figure 1:**
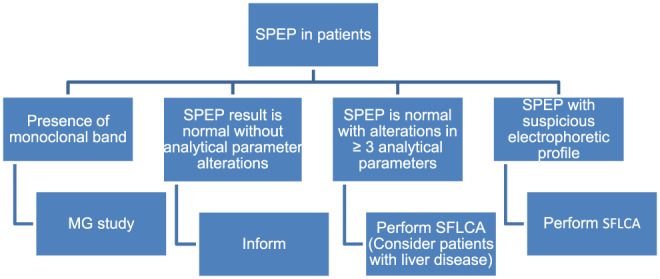
Proposed working algorithm. SPEP: serum protein electrophoresis; MG: monoclonal gammopathy; SFLCA: serum free light chain assay.

Consecutive patients referred for SPE examination and exhibiting two or more suspicious alterations (Table 2), or referred for UIFE or SIFE without a previous diagnosis of MG were prospectively selected over a one-month period using the algorithm displayed in Figure 1 **.** Of the 261 patients, 48% were referred for SPE only, whereas 3% were referred for UIFE only. The remainder of patients were prescribed a combination of several tests (Table 3). At six months, the medical records of these patients were reviewed to determine whether initial suspicion of MG was maintained, and 28 diagnoses were confirmed.

**Table 3:** Type and frequency of analytical profiles related to the detection of monoclonal components.

Profile^a^	Requests, n (%)^b^
Only UIFE	8 (3%)
UIFE + SPEP	42 (16%)
UIFE + SPEP + SIFE	18 (7%)
Only SPEP	125 (48%)
SPEP + SIFE	68 (26%)
Total	261 (100%)

^a^UIFE, urine protein immunofixation electrophoresis; SPEP, serum protein electrophoresis; SIFE, serum protein immunofixation electrophoresis. ^b^Frequency of each type of request received in the laboratory.

The retrospective study showed that the combination of SPE and SFLCA was the most simple and sensitive protocol for the detection of new monoclonal proteins. Applied to the 261 patients, this algorithm prospectively identified all MG diagnoses (27 of 28), except for a MGUS. If the algorithm proposed was adopted, requests for SIFE and UIFE examination would decrease by 68 and 60%, respectively (Table 4). Otherwise said, SIFE and UIFE would only be performed in the presence of a positive SPE + SFLCA test result or on suspicion of AL. The incorporation of this protocol would save 15.6 h of work (considering 50 min to analyze 20 serum samples for SFLCA and 4 min for validation).

**Table 4:** Impact of the implementation of the new screening protocol based on SPEP + FLCA.

Diagnostic test	No. of requests received	Δ No. of tests performed with the new protocol (SPEP + FLC)	Δ Time (h) devoted to perform the test with the new protocol	Δ Time (h) devoted to validation with the new protocol
UIFE	68	−40	−6.4	−0.9
SIFE	86	−58	−19.3	−1.4
SPEP	253	+8	+0.3	+0.2
SFLCA	0	+261	+10.9	+1.0
Total	407	+171	−14.5	−1.1

Number of requests per test during the prospective study period. This number exceeds that of patients, as more than one test was ordered in 49% of patients.

Frequency of UIFE and SIFE requests was based on the fact that only the samples of the 261 patients with a positive test for MG (28) or suspicion of AL (0) would undergo the test; SPEP and SFLCA would be performed in all the patients studied (261).

## Discussion

Diagnosis of malignant MG may be delayed 6 months from first presentation of symptoms in primary care to final diagnosis [9]. This delay is due to the non-specificity of symptoms and low level of suspicion because of the relative low incidence of this disease. In this setting, screening tests are poorly effective and entail a heavy workload for laboratories. With the twofold purpose of improving the efficacy of screening tests and raise suspicion of MG, this study was performed:

1. To identify the test combination with the highest sensitivity that leads to a lighter workload for the laboratory.
2. To determine a biochemical profile that allowed us to design a screening algorithm.

In line with other authors [4], [5], [6], [7], [8], [9], [10], this study demonstrates the high sensitivity of a screening panel based on SPE and SFLCA examination in serum for the detection of symptomatic MG. However, the use of this panel alone does not improve the efficacy of the entire screening process by itself. It is necessary to establish the setting where the use of this protocol is justified despite a low level of suspicion. The results of this study demonstrate that alterations in three or more biochemical parameters should raise suspicion of MG and would justify the use of our screening panel (SPE + SFLCA). This suspicion would be grounded on the fact that co-occurrence of these alterations is significantly less frequent in patients without MM (<2%) as compared to patients with MM or AL (61%). To the best of our knowledge, this is the first case-control study to assess the incidence of co-occurrence of biochemical alterations in MG. A similar approach was adopted by Goldschmidt et al. [8], who selected a control population of patients with back pain. The authors report that backache with concomitant fatigue, weight loss, and laboratory test alterations are suggestive of MM. The algorithm proposed by these authors may be useful for the clinician, but not for the laboratory, as data on this symptomatology (i. e. back pain, weight loss) rarely are reported on test request forms. Co-occurrence of several alterations or symptoms has also been investigated in previous studies, although separately and in diseased populations [11], [12]. This type of analysis has a low PPV and specificity for the detection of MM. Thus, although a high percentage of patients with MM exhibit some of these alterations (e. g. anemia or renal insufficiency), these signs are also found in other much more frequent diseases. A high percentage of patients with MM experience back pain, but only 1% of the population with this symptom has a malignant disease [13]. A PPV of 80% is widely accepted as the lower cut-off for a variable to be considered to have sufficient predictive value to be used in daily practice. In our study, although the presence of five of the six variables was significantly more frequent in the MM population, in agreement with previous studies [8], only TP>8.7 g/dL and bone damage had a PPV>80%. However, the individual frequency of these variables in the diseased population was <50%, which means that the level of suspicion of MM would not increase significantly. For this reason, co-occurrence of several symptoms in a single patient was analyzed to increase PPV and NPV.

An increasing number of hospital laboratories are building alarm algorithms based on common laboratory tests, which has resulted in the development of more specific and sensitive screening protocols. These algorithms facilitate early diagnosis, reduce laboratory workload, and save patients the inconvenience of travelling to hospital.

However, although promising results have been obtained, the ratio of cases to controls (54:120) in this retrospective study does not reflect the real ratio, as in real practice the prevalence of MM is 4–6 cases per 100,000. This limitation means that the PPV and NPV values obtained in our study need to be interpreted cautiously. Nevertheless, prospective analysis of 261 patients demonstrates that the use of the SPE + SFLCA screening algorithm in a setting of clinical or analytical suspicion based on the co-occurrence of symptoms would significantly reduce the workload of laboratories and have a higher diagnostic sensitivity as compared to a single test (SPE or UIFE). The purpose of this study is not to provide a health economics model, but to increase the reliability of screening methods.
